# A Scale for Older adults’ decisional balance regarding physical ACTIVity (SO-ACTIV): development and validation in a French sample

**DOI:** 10.3389/fpubh.2026.1728788

**Published:** 2026-02-24

**Authors:** Camille Giaufer, Meggy Hayotte, Raphaëlle Ladune, Raphaël Zory, Frédéric Prate, Fabienne d’Arripe-Longueville

**Affiliations:** 1 Université Côte d’Azur, LAMHESS, Nice, France; 2 Department of Geriatric Medicine, Clinique Gériatrique de Soins Ambulatoires, Université Côte d’Azur, Centre Hospitalier Universitaire de Nice, Nice, France

**Keywords:** barriers, facilitators, psychometric validation, senior, transtheoretical model

## Abstract

**Background:**

Despite its health benefits, physical activity declines with age. The transtheoretical model posits that individuals’ choice to be active depends on their perceived decisional balance (i.e., facilitators weight against barriers). Some of these factors are age-specific and extend across multiple socioecological levels (intrapersonal, interpersonal, environmental, organizational). Yet, existing decisional balance measures overlook age-related concerns, lack theoretical grounding, or fail to reflect the multilevel socioecological structure. To address these gaps, we developed and validated the Scale for Older adults’ decisional balance regarding physical ACTIVity (SO-ACTIV).

**Methods:**

Following established steps for scale development, an online survey was completed by 452 French-speaking older adults. Confirmatory factor analyses tested different models. Reliability was examined through internal consistency and 2-week test–retest. Construct validity was assessed through correlations with motivation toward health-oriented physical activity, a decisional-balance measure for exercise in adults, and a self-reported physical activity level score. Known-groups validity was tested across stages of change.

**Results:**

From an initial pool of 55-item, expert review retained 35-item divided into two overarching factors (facilitators, barriers) and four domains (intrapersonal, interpersonal, environmental, organizational). Confirmatory factor analyses supported a final 26-item instrument structured as a second-order hierarchical model, with two upper-level factors, and four specific domains for each. This model yielded the best fit indices. Internal consistency ranged from acceptable to excellent across domains and factors, and test–retest reliability was mainly demonstrated. Convergent validity was supported: facilitators correlated positively with self-determined forms of motivation and with physical activity, and negatively with amotivation; barriers showed the opposite patterns. Concurrent validity was attested by correlations between facilitators and pros, and between barriers and cons. Known-groups validity was supported by higher facilitator scores and lower barrier scores at action/maintenance stages compared with earlier stages of change.

**Conclusion:**

SO-ACTIV is a brief, theory-based instrument that captures facilitators and barriers to physical activity among French-speaking older adults across intrapersonal, interpersonal, environmental, and organizational levels. Its strong psychometric qualities support its use in future research and to guide targeted counseling and interventions in physical activity.

## 1 Introduction

Physical inactivity represents a major public health concern, especially among older adults, for whom it is associated with increased risks of mortality, chronic diseases, frailty, and loss of autonomy (1). In contrast, regular physical activity (PA) is widely recognized as a cornerstone of healthy aging. It is associated with reduced cardiovascular disease risk, a major contributor to mortality worldwide (2), and is widely recommended as a first-line lifestyle strategy for clinical prevention and management (3, 4). Accordingly, the World Health Organization (WHO) recommends that adults engage in 150 to 300 min of moderate-intensity aerobic PA per week, complemented by muscle-strengthening and flexibility exercises (5). Despite these well-established benefits and recommendations, older adults remain the least physically active demographic group worldwide (6). This persistent gap highlights the need for more effective and targeted interventions, which requires a nuanced and deep understanding of the factors that influence older adults’ engagement or disengagement in PA.

The determinants of PA are multifactorial, interconnected, and operate across multiple levels, as conceptualized within the socioecological framework (7). Among older adults, barriers to PA (i.e., factors that limit or restrict participation in PA) have been reported across levels (8), including (a) intrapersonal factors such as pain, fatigue, or fear of falling (9–11); (b) interpersonal factors such as lack of support, or isolation (10, 12); (c) environmental factors such as poor access to facilities (10, 12, 13); and (d) organizational factors such as limited adapted programs (9, 10, 12, 14). Conversely, facilitators of PA (i.e., factors that promote or enable engagement in PA) have also been identified across these same levels (8). At the intrapersonal level, key motivators include perceived health benefits (e.g., disease prevention, pain reduction), enjoyment, or increased physical and mental wellbeing (10, 12, 15, 16). At the interpersonal level, encouragement from relatives, peers, health professionals, or even pets, as well as the social aspect of group classes, are frequently cited (10, 16, 17). At the environmental level, access to safe, nearby walking areas and adapted exercise spaces (e.g., at-home options) supports engagement (10, 12, 13). Finally, at the organizational level, flexible schedules, low-cost or free programs, adapted content, and qualified instructors are key enablers (10, 12, 18). Despite a growing body of research on the determinants of PA in older adults, most studies remain descriptive or exploratory, with limited theoretical foundations (10).

The Transtheoretical Model (TTM) (19) offers a dynamic, stage-based framework for understanding health behavior change. According to this model, individuals progress through five stages (precontemplation, contemplation, preparation, action, and maintenance) based on their readiness to adopt a new behavior. This progression is influenced by several key constructs, including decisional balance (DB) (i.e., weighing perceived benefits and barriers), self-efficacy (confidence in one’s ability to change), cognitive and behavioral processes of change, and temptation (the expected urge to lapse in difficult situations). Among these constructs, DB, derived from decision-making theory (20), plays a pivotal role. It refers to the internal evaluation of perceived pros and cons that shape a person’s intention to act. According to the TTM, behavioral change occurs when the perceived benefits begin to outweigh the perceived barriers, making DB a particularly relevant construct for understanding older adults’ engagement or disengagement in PA.

Although several instruments assess perceived barriers and facilitators of PA, key gaps remain regarding theoretical grounding, age-specificity, and socioecological framework coverage. Some widely used measures assess the DB constructs (i.e., pro/cons) but are not grounded in the TTM, such as the *Exercise Benefits/Barriers Scale (EBBS)* and its French adaptation (21, 22), which are based on the Health Promotion Model, or the *Outcome Expectations for Exercise scale* (23, 24), originally developed in English, which is based on the Outcome Expectancy theory. Among TTM-based instruments, such as the *Decisional Balance Scale for Exercise* and its French adaptation (25, 26), these tools were not specifically developed for older adults. When considering scales designed for older adults, some, such as the *Aging Stereotypes and Exercise Scale* (27), which has been validated in French focus primarily on intrapersonal factors and does not cover the multiple levels of the socio-ecological framework. Other measures, including the *Inventory of Physical Activity Barriers* (28, 29) and the *Physical Activity Barriers Scale for the Elderly* (PABSE) (30), are explicitly designed for older adults and address multiple levels of the socio-ecological framework; however, they are not available in French (respectively available in English and Turkish) and assess barriers only. Taken together, and to the best of our knowledge, no French-validated TTM-based DB tool simultaneously captures barriers and facilitators across the socioecological levels while addressing older adults’ age-related concerns. This gap underscores the need for a comprehensive instrument that integrates barriers and facilitators across socioecological levels, within a unified DB construct, and is tailored to older adults.

To address these gaps, the present study aimed to develop and validate the Scale for Older adults’ decisional balance regarding physical ACTIVity (SO-ACTIV). Anchored in the DB construct of the TTM and informed by a socioecological perspective, SO-ACTIV is specifically designed to assess the perceived barriers and facilitators encountered by older adults across intrapersonal, interpersonal, environmental, and organizational domains.

## 2 Methods

### 2.1 Study design and ethical considerations

This research employed a cross-sectional survey design following recommended steps for scale development and validation (31, 32). Accordingly, the validation process was divided into two phases. Phase 1 involved item generation, initial content validation, and pretesting. Phase 2 consisted of scale evaluation, including tests of dimensionality, reliability and validity. Data were collected online between January and March 2025 using LimeSurvey (version 3.17.3; LimeSurvey, CE). All participants read a detailed study information sheet and could ask questions before taking part. Participation required electronic informed consent by clicking on “I agree to participate”. Participants could withdraw at any time without penalty. Data were collected anonymously to ensure confidentiality and privacy. This study was approved by the Université Côte d’Azur Ethics Committee (No. 2025–025).

### 2.2 Phase 1: Item and scale development

#### 2.2.1 Identification of domain and item generation

SO-ACTIV was developed as an original instrument to assess perceived facilitators and barriers to regular PA among adults aged 55 and older, based on the DB construct (20). Its development was guided by a socioecological framework, which conceptualizes PA behavior as shaped by multilevel factors across intrapersonal, interpersonal, environmental, and organizational domains (7, 33, 34). A preliminary step conducted by the research team in November 2024 (PubMed, ScienceDirect, SAGE) consisted of an examination of the current literature (e.g., “physical activity” OR “exercise”; “barriers” OR “facilitators” OR “motivators”; “older adults” OR “elderly” OR “aged”). A total of 77 studies reporting factors facilitating or limiting PA engagement in older adults were identified (e.g., (8–17)). Based on these findings, the research team extracted recurring determinants reported in older adults and organized them into a structured content map crossing (a) DB polarity (barriers vs. facilitators) and (b) the four socioecological domains (intrapersonal, interpersonal, environmental, organizational). This content map served as the blueprint for item writing, ensuring coverage of each domain for both barriers and facilitators. Items were generated directly in French based on these categories of factors for each dimension (no translation/back-translation process was conducted). Existing validated instruments were consulted as wording templates to standardize phrasing and format (21, 24–27, 29, 30). For example, facilitator items were framed as concrete reasons for engaging in PA (“I engage in physical activity regularly because…”), whereas barrier items were framed as concrete reasons for non-engagement (“I do not engage in physical activity regularly because…”). Wording was refined to avoid double-barreled items and to use age-appropriateness, and relevance for older adults.

#### 2.2.2 Evaluation by experts and target population

Ten experts in PA, psychometrics, and aging (M_age_ = 33.8 ± 9 years; 80% PhD; 60% women) from the Empowerment and Social Design group of the ANR Pré.S.age consortium were consulted. Experts were defined as researchers, PhD students, or practitioners with at least a master’s degree and documented experience in (a) promoting PA in older adults and/or (b) scale development. Experts were asked to rate each item for both relevance to the construct, and clarity of formulation, using a 7-point scale (1=“not at all relevant/clear”, to 7 = “completely relevant/clear”). In parallel, a convenience sample of twelve older adults (M_age_ = 60.7 ± 3.7 years; 50.0% women) assessed item clarity. These participants were recruited through the research team’s networks (eligibility criteria: French-speaking; ≥55 years; voluntary participation). After the initial ratings, item-level summaries (means and anonymized comments) were compiled. Items were flagged for further review when mean relevance and/or clarity ratings were <6.0 and/or when recurrent comments suggested ambiguity or redundancy. Flagged items were then discussed iteratively with the expert panel using a structured consensus approach: proposed edits were derived from qualitative feedback, circulated among panel members, and refined until agreement was reached on whether to retain, revise, or delete each item. The clarity of the resulting pool of items was subsequently checked in a second convenience sample of 22 older adults (M_age_ = 67.0 ± 10.7 years; 59.1% women), recruited using the same procedure.

### 2.3 Phase 2: Scale evaluation

Phase 2 consisted of scale evaluation including tests of dimensionality, tests of reliability and tests of validity. The main sample with all participants was used for the dimensionality tests. Since additional measurements were needed for some reliability and validity tests, specific subsamples from the main sample were used.

#### 2.3.1 Participants and sample size

Eligibility criteria were as follows: (a) French-speaking adults and (b) aged 55 years or older. Exclusion criteria were limited to non-provision of the electronic informed consent and incomplete data. A total of 475 French-speaking adults aged 55 years and older completed the online survey. To enhance sample diversity, participants were recruited through three sources: a health conference in France (*n* = 45), and two established research platforms, namely Crowdpanel (*n* = 157), and Prolific (*n* = 273). Participants from Crowdpanel and Prolific platforms received financial compensation (platform-standard rates), whereas conference participants completed the survey on a voluntary basis and were provided with information about free PA programs. A multi-step data-quality screening protocol adapted from (35) was applied to identify low-quality responses: cases with >20% missing responses (*n* = 14), duplicates (*n* = 2), and respondents flagged by a combined speed and straight-lining indicator (very fast completion with very low within-scale variability) (*n* = 7) were excluded. The final baseline sample was composed of 452 responses (45 from health conference, 150 from Crowdpanel, and 257 from Prolific). This main sample was used for most of the psychometric analyses (dimensionality, internal consistency, known-groups validity). A subsample from Crowdpanel (*n* = 150) completed additional validated instruments for construct validity. For test–retest reliability, 82 participants were recontacted from Prolific after 2 weeks to complete the questionnaire again (4 were excluded due to incomplete or inconsistent follow-up). The test–retest subsample comprised 78 participants.

#### 2.3.2 Measures

##### 2.3.2.1 Scale for Older adults’ decisional balance regarding physical ACTIVity (SO-ACTIV)

SO-ACTIV assesses perceived facilitators and barriers to regular PA among adults aged 55 and older. Participants rated 35 items (18 facilitators; 17 barriers), on a 5-point Likert scale ranging from 1 (“Strongly disagree”) to 5 (“Strongly agree”). Facilitator items were phrased as reasons for engaging in PA (e.g., “I engage in physical activity regularly because…”), while barrier items reflected reasons for not engaging (e.g., “I do not engage in physical activity regularly because…”). The instrument was administered to the main sample (*n* = 452) and a second time to the test–retest subsample (*n* = 78).

##### 2.3.2.2 Decisional balance for physical activity

DB for PA was measured with a validated French adaptation (25) of the Decisional Balance for Exercise scale, based on the TTM (19, 20, 36). It comprises 16 items divided into two subscales (Pros/Cons; 5-point Likert). The DB for PA scale was completed by the Crowdpanel subsample for convergent validity analysis. Internal consistency in the present study was excellent (i.e., *α* = 0.93) for the pros subscale, and very good (*α* = 0.82) for the cons subscale.

##### 2.3.2.3 Self-reported physical activity score

PA was measured using the “Score d’Activité Physique de Dijon” (37), a 9-item self-report measure evaluating weekly PA in older adults. It categorizes respondents into three levels of activity: (a) low, (b) moderate, or (c) high. The PA score was assessed in the Crowdpanel subsample (n = 150) to assess convergent validity.

##### 2.3.2.4 Motivation towards health-oriented physical activity

The “*Échelle de Motivation pour l’Activité Physique à des fins de Santé*” (38) is a French developed instrument grounded in Self-determination theory (39). It comprises 18 items assessing six types of motivation, each rated on a 7-point Likert scale. In the present study, internal consistency across subscales was satisfactory to excellent (Cronbach alphas ranged from 0.81 to 0.93). The scale was administered to the Crowdpanel subsample (*n* = 150) to assess convergent validity.

##### 2.3.2.5 Physical activity stages of change

Stages of change were assessed with the standard four-item yes/no algorithm to classify participants into the five TTM stages (40, 41). The instrument was administered to the full sample (*n* = 452) and used to test known-groups validity. In previous study, this questionnaire demonstrated adequate reliability (*κ* = 0.78, 2-week test–retest; 39).

##### 2.3.2.6 Demographic data

Participants were asked to report basic demographic information, including their gender, age, and highest level of education. To maintain full anonymity and reduce identifiability risk, no additional sociodemographic variables (e.g., income, employment status, marital status, ethnicity) were collected.

#### 2.3.3 Data analysis

All analyses were conducted with IBM SPSS Statistics and Amos (version 27.0; IBM) software. Prior to conducting parametric tests, the assumption of normality was assessed by examining skewness and kurtosis (42).

##### 2.3.3.1 Tests of dimensionality

To evaluate the dimensionality of SO-ACTIV, a series of confirmatory factor analyses was conducted using structural equation modeling. Based on the DB framework and the socioecological perspective underlying item development, we hypothesized that the items would reflect two correlated higher-order dimensions (barriers and facilitators), each expressed across four domains (intrapersonal, interpersonal, environmental, organizational). Because SO-ACTIV was developed from this explicit, theory-driven *a priori* structure, dimensionality was examined directly using confirmatory factor analysis rather than exploratory analyses, as recommended when the factor structure can be specified in advance (43–45). This approach is also consistent with previous decisional balance scale validations relying on confirmatory testing of theoretically specified multidimensional structures (e.g., 20, 45, 46). First, scale was refined to improve parsimony, reliability and overall fit. Item reduction followed predefined rules aligned with scale-validation guidance (31, 48) to reduce redundancy, ambiguity, and item’s insufficient statistical contribution. Items with standardized factor loadings below 0.50, substantial standardized residuals, or content redundancy were candidates for removal, while ensuring to preserve at least three items per factor and coverage of the four socioecological domains (49).

A set of *a priori* theoretical models was specified based on established conceptual frameworks to examine alternative hypotheses regarding the latent structure (50). The following models were tested: (a) a unidimensional model (model 1) representing a unique and undifferentiated factor of DB; (b) a two-factor correlated model (model 2) distinguishing perceived barriers and facilitators; (c) an eight-factor correlated model (model 3) with four specific domains (i.e., intrapersonal, interpersonal, environmental, and organizational) for barriers and four symmetrical domains for facilitators; (d) a hierarchical model (model 4) in which the eight domains are grouped into two correlated second-order factors (barriers and facilitators). Model fit was assessed using commonly recommended fit indices (48), including: (a) the chi-square statistic and its ratio to degrees of freedom (*χ*^2^/df; values <3.00 considered acceptable); (b) the root mean square error of approximation (RMSEA; values ≤0.08 considered acceptable fit); (c) the comparative fit index (CFI), and Tucker-Lewis index (TLI), with values ≥0.90 indicating good fit; and (d) the goodness of fit index (GFI), and adjusted goodness of fit index (AGFI), for which values >0.80 indicate acceptable fit.

##### 2.3.3.2 Tests of reliability

Internal consistency of the SO-ACTIV was assessed using Cronbach’s alpha and McDonald’s omega coefficients. Reliability was evaluated at two levels: (a) the two global dimensions (barriers and facilitators), and (b) the four domain-specific subscales within each dimension (intrapersonal, interpersonal, environmental, and organizational). Cronbach’s alpha values between 0.70 and 0.79 were considered acceptable, and values ≥0.80 were considered good (51). McDonald’s omega values ≥0.70 were considered to reflect satisfactory internal consistency (52).

Temporal stability was examined over a 2-week interval in a subsample of 78 participants. Paired sample t-tests were used to compare scores between time 1 and time 2. No significant differences indicate good stability over time (53).

##### 2.3.3.3 Tests of validity

Construct validity was assessed through concurrent and convergent approaches. Bivariate Pearson correlations were computed between the SO-ACTIV scores (global barriers and global facilitators dimensions) and theoretically related measures: (a) pros and cons from the DB for PA (25); (b) self-reported PA score (37); and (c) different forms of motivation (38). Pearson correlation values between 0.10 and 0.30 were considered small, 0.30 to 0.50 moderate, 0.50 to 0.70 large, and above 0.70 very large (54). Positive correlations were expected between pros and facilitators and between cons and barriers. Self-reported PA scores were expected to correlate positively with facilitators and negatively with barriers. In line with self-determination theory (39), positive correlations were anticipated between facilitators and the most self-determined forms of motivation (i.e., intrinsic motivation, extrinsic motivation with integrated regulation and identified regulation), and negative correlations with the least self-determined forms (i.e., amotivation, extrinsic motivation with external regulation). Conversely, barriers were expected to correlate negatively with the most self-determined forms of motivation, and positively with amotivation and external regulation.

Known-groups validity (31) was assessed by comparing participants’ DB scores (barriers and facilitators) across three predefined behavioral stages of PA engagement based on the TTM (precontemplation/contemplation, preparation, and action/maintenance) (26). The objective was to determine whether SO-ACTIV could effectively distinguish between these groups. It was hypothesized that perceived barriers would decrease, and perceived facilitators would increase with progression through more advanced stages of change. A multivariate analysis of variance (MANOVA) was conducted to assess the overall effect of stage on the combined scores for global barriers and facilitators. Subsequently, univariate ANOVAs were performed for each dimension (barriers and facilitators) to examine specific differences, followed by post-hoc comparisons to determine significant pairwise differences between stages.

## 3 Results

### 3.1 Phase 1: Item and scale development

#### 3.1.1 Identification of domain and item generation

Based on the 77 studies identified by the literature review conducted and on similar existing scales, an initial pool of 55 items was developed and classified into four domains (intrapersonal, interpersonal, environmental, organizational). The development of new items improved domain coverage.

#### 3.1.2 Evaluation by experts and target population

Experts and older adults examined the scale for relevance and clarity. Scores below 6.0 were flagged for further discussions and refinement with experts. Among the initial pool of 55 items, 25 items were removed, 8 were revised, and 5 were added in alternative formulations to examine the best version of a single item. The final draft contained 35 items (18 facilitators; 17 barriers). The 35-item SO-ACTIV was pretested. Participants rated each item’s clarity, with mean clarity scores ranging from 6.4 to 7.0. No formulation or relevance issues were reported, so no further modifications were made prior to large-scale administration.

### 3.2 Phase 2: Scale evaluation

#### 3.2.1 Participants’ characteristics

The main sample included 160 men (35.4%) and 292 women (64.6%), with ages ranging from 55 to 85 years. Participants’ characteristics of the main sample (*n* = 452) and subsamples (*n* = 78 for test–retest; *n* = 150 for construct validity) are presented in Table 1.

**Table 1:** Participants’ characteristics of the main sample (*n* = 452) and specific subsamples used for test–retest analysis (*n* = 78) and construct validity analysis (*n* = 150).

Characteristics	Group	Main sample (*n* = 452)		Test–retest subsample (*n* = 78)		Construct validity subsample (*n* = 150)	
		*n*	*%*	*n*	*%*	*n*	*%*
Gender	Women	292	64.6	50	64.1	82	54.7
	Men	160	35.4	28	35.9	68	45.3
Age (years)	55–64	292	64.6	54	69.2	109	72.7
	65–74	131	29.0	20	25.6	38	25.3
	75–84	28	6.2	4	5.1	3	2.0
	≥85	1	0.2	0	0.0	0	0.0
Level of education (years)	<12	63	13.5	7	9.0	35	23.3
	12	139	29.0	16	20.5	71	43.3
	15	108	23.9	15	19.2	18	12.0
	≥17	142	33.6	40	51.3	26	21.3

Level of education: <12: French junior High School Diploma, Professional Aptitude Certificate, Professional Studies Certificate, 12: High School Diploma; 15: Bachelor’s degree; ≥17: Master’s degree or PhD.

#### 3.2.2 Tests of dimensionality

In the first step, structural equation modeling was conducted on the 35-item version of SO-ACTIV using an eight-factor correlated model. The model yielded moderate fit indices [*χ*^2^(532) = 1538.85, *p* < 0.001; *χ*^2^/df = 2.89; CFI = 0.86; TLI = 0.84; RMSEA = 0.07 [90% CI: 0.06–0.07]; GFI = 0.83; AGFI = 0.80]. This version included alternative formulations of some items, which were subsequently removed. Following pre-specified item-reduction criteria and review by an expert-committee, nine items were deleted. Seven items were removed due to redundancy or content overlap with retained items. For example, the item “Not feeling comfortable in group activities” (BInter; loading = 0.59) overlapped conceptually with the retained item “I feel embarrassed or intimidated by the presence of other participants,” and the item “Finding my environment unsafe” (BEnv; loading = 0.55) was removed in favor of the more specific retained item “My practice places are not safe (uneven sidewalks, risk of falls…)”. Two additional items were excluded due to low standardized factor loadings: “Other commitments limit me (volunteering, family, medical appointments…)” (BOrga; loading = 0.44) and “Being able to exercise from home” (FEnv; loading = 0.27). This process resulted in a refined 26-item version of the scale (see Table 2).

**Table 2:** The 26-item version of the Scale for Older adults’ decisional balance regarding physical ACTIVity (SO-ACTIV).

Dimensions	Items
Facilitators	Je pratique une activité physique régulièrement parce que… *[I engage in physical activity regularly because …]*
FIntra 1	…J’améliore ma condition physique *[I improve my physical fitness]*
FIntra 2	…Je me sens mieux *[I feel better]*
FIntra 3	…Je prends plaisir à pratiquer *[I enjoy being physically active]*
FIntra 4	…Je veux préserver ma santé *[I want to preserve my health]*
FInter 1	…Je rencontre d’autres personnes *[I meet other people]*
FInter 2	…J’ai quelqu’un avec qui pratiquer *[I have someone to exercise with]*
FInter 3	…J’apprécie l’ambiance créée par le prof/l’intervenant *[I enjoy the atmosphere created by the instructor]*
FEnv1	…Les offres de pratiques sont proches de chez moi *[Physical activity options are available near where I live]*
FEnv2	…Les lieux de pratique sont sécurisés *[The exercise locations are safe]*
FEnv3	…L’accès à des lieux de pratique est. adapté *[Access to practice areas is adapted]*
FOrga 1	…Il y a régulièrement des informations sur les programmes d’activité physique disponibles *[There is regular information on the physical activity programs available]*
FOrga 2	…Il existe des programmes conçus en fonction de mes besoins *[There are programs tailored to my needs]*
FOrga 3	…Les horaires de pratique sont flexibles ou adaptés à mon emploi du temps *[Exercise times are flexible or adapted to my schedule]*
Barriers	Je ne pratique pas une activité physique régulièrement parce que… *[I do not engage in physical activity regularly because…]*
BIntra 1	…Je suis fatigué(e) *[I feel tired]*
BIntra 2	…Je ressens des douleurs physiques *[I have physical pain]*
BIntra 3	…J’ai peur de me faire mal *[I’m afraid of getting hurt]*
BIntra 4	…Je pense que mes capacités physiques sont insuffisantes *[I think my physical abilities are not good enough]*
BInter 1	…Je n’ai pas de soutien de la part de mon entourage personnel et/ou professionnel *[I do not get support from people around me (personal or professional)]*
BInter 2	…Je n’ai personne avec qui pratiquer *[I do not have anyone to practice with]*
BInter 3	…Je me sens gêné(e) ou intimidé(e) par la présence d’autres participants *[I feel uncomfortable or intimidated by the presence of other participants]*
BEnv 1	…Mes lieux de pratique ne sont pas sécurisés (trottoirs inégaux, risques de chutes…) *[The places where I could exercise are not safe (uneven sidewalks, risk of falling, etc.)]*
BEnv 2	…L’accès à mes lieux de pratique est. difficile (distance, stationnement) *[Access to my practice areas is difficult (distance, parking)]*
BEnv 3	…Mon lieu de vie est. isolé *[I live in an isolated area]*
BOrga 1	…Les offres de pratiques proposées sont trop chères *[The available activities are too expensive]*
BOrga 2	…Il n’existe pas d’offre de pratique qui me convienne *[There is no practice offer that suits me]*
BOrga 3	…Les offres existantes manquent d’intervenants qualifiés/compétents *[The existing programs do not have enough qualified or skilled instructors]*

BIntra, intrapersonal barriers; BInter, interpersonal barriers; BEnv, environmental barriers; BOrga, organizational barriers; FIntra, intrapersonal facilitators; FInter, interpersonal facilitators; FEnv, environmental facilitators; FOrga, organizational facilitators. Texts in italics represents a free, non-validated English translation.

**Figure 1:**
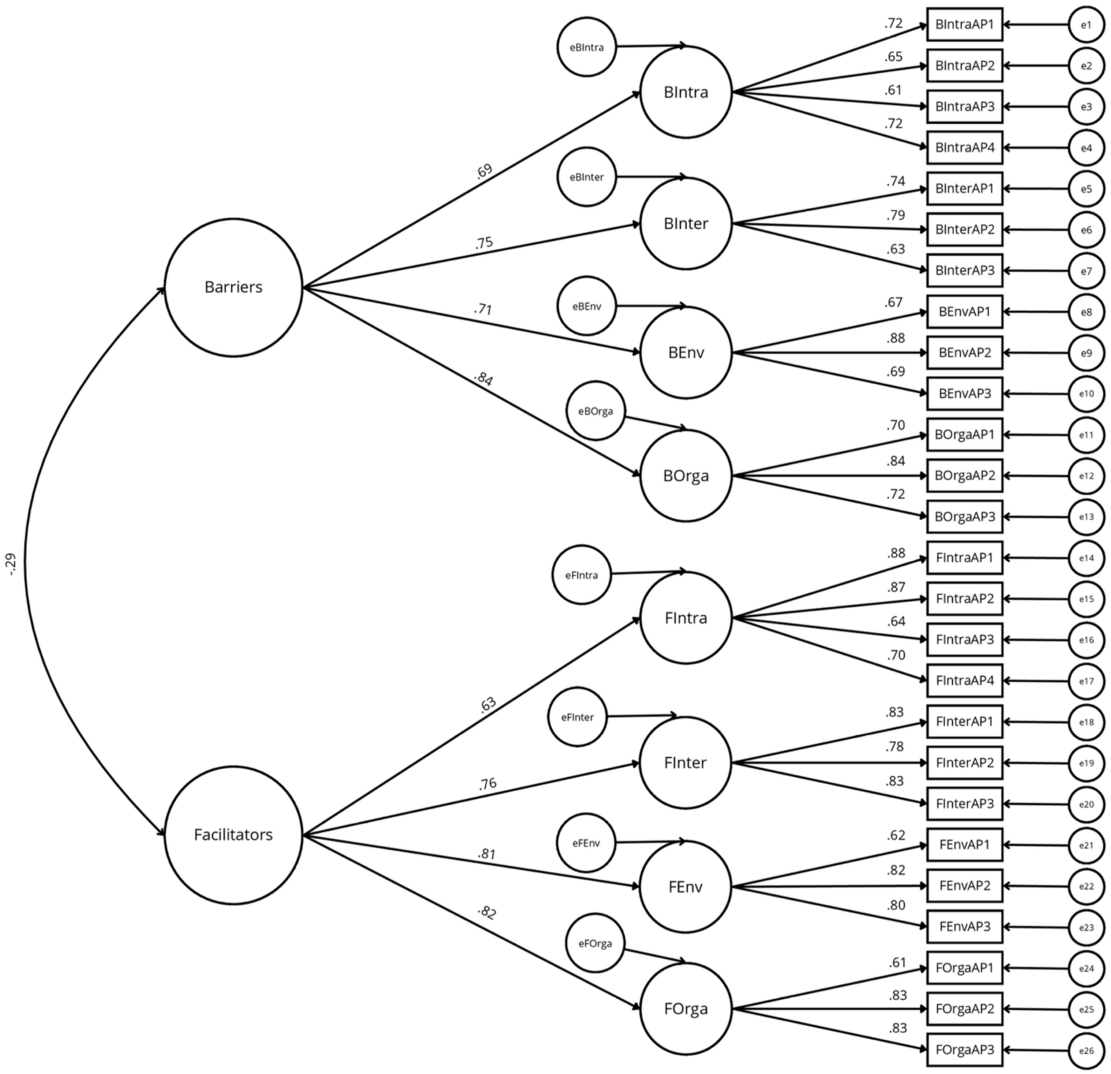
Second-order hierarchical confirmatory factor model of the SO-ACTIV (*n* = 452). B, barrier; F, facilitator; Intra, intrapersonal; Inter, interpersonal; Env, environmental; Orga, organizational.

In the second step, four pre-specified models were tested on the 26-item version of SO-ACTIV. Model fit indices are reported in Table 3. The unidimensional and two-factor models failed to meet acceptable fit criteria (CFI, TLI < 0.70; RMSEA>0.11). In contrast, both the eight-factor correlated model (Model 3) and the second-order hierarchical model (Model 4) demonstrated acceptable model fit. Although Model 3 yielded slightly better fit indices [*χ*^2^(271) = 697.70, *p* < 0.001; *χ*^2^/df = 2.57; CFI = 0.92; TLI = 0.91; RMSEA = 0.06 [0.05–0.07]; GFI = 0.89; AGFI = 0.86], Model 4, which enables the estimation of the eight socioecological domains factors (intrapersonal, interpersonal, environmental, organizational) and two second-order dimensions (barriers and facilitators), also showed good fit indices [*χ*^2^(290) = 774.85, *p* < 0.001; *χ*^2^/df = 2.67; CFI = 0.91; TLI = 0.90; RMSEA = 0.06 [0.06–0.07]; GFI = 0.88; AGFI = 0.86]. Consistent with the theoretical framework and intended scoring strategy of the instrument, Model 4 was retained to enable the computation of both domain-level scores and global barriers/facilitators scores. Standardized factor loadings for this model are presented in Figure 1.

**Table 3:** Fit indexes of the different models examined by the confirmatory factor analysis (*n* = 452).

Models	X^2^ (df)	RMSEA [90% confidence interval]	GFI	AGFI	CFI	TLI	ΔX^2^
Uni-dimensional (model 1)	3,419.37 (299)	0.152 [0.148–0.157]	0.50	0.42	0.42	0.37	
2-factor correlated (model 2)	2,113.41 (298)	0.116 [0.112–0.121]	0.70	0.64	0.66	0.63	1305.96 (1)^***^
8-factor correlated (model 3)	697.70 (271)	0.059 [0.054–0.065]	0.89	0.86	0.92	0.91	1415.71 (27)^***^
**Hierarchical (model 4)**	**774.85 (290)**	**0.061 [0.056–0.066]**	**0.88**	**0.86**	**0.91**	**0.90**	**77.15 (19)** ^***^

*χ*^2^, Chi-squared; df, degrees of freedom; RMSEA, root mean square error of approximation; 90% CI, 90% confidence interval of RMSEA; GFI, goodness-of-fit index; AGFI, adjusted goodness-of-fit index; CFI, comparative fit index; TLI, Tucker-Lewis index of adjustment. ***p < 0.001. The selected model is in bold.

#### 3.2.3 Tests of reliability

Internal consistency was computed for each of the eight domain subscales and the two global factors using both Cronbach’s alpha (*α*) and McDonald’s omega (*ω*). As shown in Table 4, all dimensions demonstrated acceptable to excellent reliability (α ranging from 0.75 to 0.85; ω from 0.77 to 0.86).

**Table 4:** Internal consistency of the dimensions of the Scale for Older adults’ decisional balance regarding physical ACTIVity (*n* = 452).

Dimensions of the SO-ACTIV questionnaire	Cronbach’s α	McDonald’s ω
Global barriers	0.77	0.78
Intrapersonal barriers	0.77	0.77
Interpersonal barriers	0.75	0.77
Environmental barriers	0.78	0.80
Organizational barriers	0.79	0.80
Global facilitators	0.79	0.80
Intrapersonal facilitators	0.84	0.85
Interpersonal facilitators	0.85	0.86
Environmental facilitators	0.77	0.78
Organizational facilitators	0.79	0.80

Test–retest over a 2-week interval (n = 78) showed no significant mean differences between T1 and T2 across any SO-ACTIV dimension (all *p* > 0.05; see Table 5), supporting the temporal stability of the instrument.

**Table 5:** Temporal stability of the dimensions of the Scale for Older adults’ decisional balance regarding physical ACTIVity over a 2-week interval (*n* = 78).

SO-ACTIV’s dimensions	T1 *M* (SD)	*α*	*ω*	T2 *M* (SD)	*α*	*ω*	*t*	*p*
Global barriers	2.24 (0.74)	0.80	0.80	2.20 (0.81)	0.84	0.84	0.49	0.62
Intrapersonal barriers	2.46 (0.89)	0.68	0.68	2.41 (1.00)	0.81	0.81	0.44	0.66
Interpersonal barriers	2.08 (1.11)	0.78	0.80	2.09 (0.94)	0.72	0.75	−0.12	0.90
Environmental barriers	2.01 (1.01)	0.81	0.83	2.03 (0.96)	0.79	0.81	−0.20	0.84
Organizational barriers	2.13 (0.96)	0.81	0.81	2.25 (1.02)	0.80	0.84	−1.07	0.29
Global facilitators	3.93 (0.62)	0.78	0.81	3.87 (0.63)	0.73	0.76	1.04	0.30
Intrapersonal facilitators	4.42 (0.54)	0.72	0.73	4.41 (0.55)	0.76	0.76	0.20	0.84
Interpersonal facilitators	3.51 (1.10)	0.86	0.86	3.44 (1.09)	0.85	0.86	0.64	0.52
Environmental facilitators	3.83 (0.75)	0.64	0.71	3.81 (0.80)	0.75	0.77	0.32	0.75
Organizational facilitators	3.94 (0.71)	0.69	0.75	3.82 (0.84)	0.80	0.84	1.34	0.18

*M* (SD), Mean (Standard Deviation); *α*, Cronbach’s alpha; *ω*, McDonald’s omega; df = 77 for all *t*-tests. No dimension showed a significant difference between T1 and T2 (*p* > 0.05).

#### 3.2.4 Tests of validity

Pearson correlations between SO-ACTIV dimensions and DB for PA scale, self-reported PA score, and forms of motivation supported concurrent and convergent validity (see Table 6). Significant correlations were obtained in the expected directions.

**Table 6:** Construct validity results based on Pearson correlations (*n* = 150).

Construct validity measures	Barriers from the SO-ACTIV	Facilitators from the SO-ACTIV
Concurrent validity		
Barriers from the decisional balance for physical activity scale	0.45**	0.08
Facilitators from the decisional balance for physical activity scale	−0.06	0.38**
Convergent validity		
Self-reported physical activity score	−0.34**	0.44**
Intrinsic motivation	−0.26**	0.60**
Extrinsic motivation with integrated regulation	−0.34**	0.50**
Extrinsic motivation with identified regulation	−0.28**	0.62**
Extrinsic motivation with introjected regulation	−0.28**	0.53**
Extrinsic motivation with external regulation	0.21**	0.13
Amotivation	0.33**	−0.18*

SO-ACTIV, Scale for Older Adults’ decisional balance regarding physical ACTIVity. *p < 0.05; **p < 0.01.

Results of the known-groups validity analysis are presented in Table 7. The MANOVA indicated a significant overall stage effect on SO-ACTIV barriers and facilitators [Wilks’ *Λ* = 0.87, *F*(4, 896) = 15.79, *p* < 0.001]. Univariate ANOVAs showed stage differences for barriers [*F*(2, 449) = 10.65, *p* < 0.001] and facilitators [*F*(2, 449) = 26.62, *p* < 0.001]. Post-hoc tests (Tukey’s HSD) revealed that participants in action/maintenance stage reported fewer perceived barriers than those in precontemplation/contemplation and preparation, with no difference between the latter two stages. Conversely, facilitators score increased across stages, from precontemplation/contemplation to preparation, and to action/maintenance.

**Table 7:** Known-groups validity of the Scale for Older adults’ decisional balance regarding physical ACTIVity barriers and facilitators by stage of change (*n* = 452).

Stage of change	*n*	Barriers *M* (SD)	Facilitators *M* (SD)	*Post-hoc* comparisons	
				Barriers	Facilitators
1. Precontemplation/contemplation	54	2.65 (0.67)	3.03 (0.92)	1 > 3	1 < 2,3
2. Preparation	65	2.52 (0.69)	3.49 (0.73)	2 > 3	2 > 1; 2 < 3
3. Action/maintenance	333	2.22 (0.76)	3.75 (0.64)	3 < 1, 2	3 > 1, 2

*M* (SD), Mean (Standard Deviation).

## 4 Discussion

This study developed and validated SO-ACTIV, a DB scale specifically designed for adults aged 55 and older. By integrating aging-related concerns and grounding its structure in both the TTM (19) and a socioecological perspective (7, 33, 34), SO-ACTIV addresses key limitations of existing measures and captures the interplay between individual ambivalence and multilevel contextual influences.

The scale development followed recommended steps for psychometric research (31, 32). The final version comprises 26 items, structured around two overarching dimensions (facilitators and barriers), each subdivided into four domains: (a) intrapersonal (4 items), (b) interpersonal (3 items), (c) environmental (3 items), and (d) organizational (3 items). This multidimensional structure enables a comprehensive profiling of PA determinants, thereby supporting the identification of actionable drivers of PA in both clinical and public health contexts (55). Both the correlated eight-factor model and the second-order hierarchical model showed acceptable fit. Although the correlated eight-factor solution displayed slightly better fit, the second-order model was retained as the primary scoring model because of its theoretical coherence with the DB framework, and its practical scoring utility, as it allows the computation of both domain-level and global barriers/facilitators scores. This model thus allows scoring at three levels: (a) a global DB index (facilitators minus barriers), (b) separate mean scores for facilitators and barriers (based on domain averages), and (c) eight domain-specific subscales. The self-administered format of SO-ACTIV takes less than 10 min to complete, providing healthcare professionals with rapid and nuanced insights into patients’ motivational dynamics. Internal consistency ranged from acceptable to excellent, and test–retest reliability over a 2-week interval was confirmed. These findings suggest that SO-ACTIV domain scores are sufficiently coherent and stable to support their use in identifying which types of determinants (intrapersonal, interpersonal, environmental, and organizational) may be most salient for a given individual. From a clinical perspective, SO-ACTIV is a brief, practical tool that can support motivational interviewing to promote PA among older adults. Clinicians can examine which domain most hinders or facilitates PA engagement, thereby enabling more targeted counseling. For example, elevated organizational/environmental barriers may suggest prioritizing practical, access-oriented solutions (e.g., referral to adapted programs, schedule/transport support, or identifying safe places to be active), whereas strong intrapersonal facilitators may call for strategies that build on existing motivation, such as emphasizing personally meaningful benefits and preferred activities to support sustained engagement. In addition, SO-ACTIV could be used longitudinally to monitor DB among older adults and detect early signs of relapse. Regarding validity, the expected associations with motivational regulations, PA, and DB (pros/cons) support the interpretability of facilitator and barrier scores, while observed stage differences indicate that SO-ACTIV is sensitive to meaningful behavioral differences consistent with TTM predictions.

Evidence of construct validity was consistent with theoretical expectations. Facilitators’ scores correlated positively with self-determined forms of motivation (intrinsic, integrated, identified, introjected) (38) and self-reported PA (37), and negatively with amotivation. Barriers’ scores showed the inverse pattern. Concurrent validity with the DB for PA scale was domain-specific, with pros aligning with facilitators and cons with barriers (25). Known-groups validity supported the scale’s sensitivity to behavioral stage. Participants in the action/maintenance stages reported higher facilitators and lower barriers than those in earlier stages, consistent with TTM predictions. However, discrimination between precontemplation and preparation was more modest for barriers.

Despite its strengths, this study has some limitations that must be acknowledged and delineate priorities for future research. First, SO-ACTIV is a newly developed tool evaluated using a theory-driven confirmatory factor analysis model-comparison approach. Because this structure has not yet been replicated in an independent sample, future studies should seek to confirm its structural validity across different populations and contexts. In particular, although the correlated eight-factor model showed slightly better fit than the second-order solution, future research should replicate these findings and further examine the appropriateness of the higher-order representation in independent samples and across relevant subgroups before relying on global second-order scores for between-group comparisons. Second, the sample was predominantly composed of young older adults (55–64 years), many of whom were already in action/maintenance stage of change. This sample composition may limit the external validity of the findings for older-old adults (≥75 years) and for individuals in earlier motivational stages. Given the heightened benefits of PA in the oldest groups (56), further validation of SO-ACTIV in these populations represents a key next step. Third, the exclusive reliance on self-report data introduces the possibility of social desirability and over-reporting biases (57, 58). Examining convergent validity of the SO-ACTIV with more objective measures such as accelerometry would provide stronger behavioral validation (59). Fourth, the assessment of socioeconomic characteristics was limited with socioeconomic status operationalized solely through the highest level of education. This may obscure broader social inequalities related to resources, social participation, and access to supportive environments (60). Accordingly, future studies should include more comprehensive sociodemographic profiling and formally test measurement invariance across key subgroups to ensure the stability and interpretability of the scale. Fifth, participants were recruited through heterogeneous sources (health conference and research platforms), which may have introduced source-related selection effects and heterogeneity across subcohorts (61, 62). In addition, because validation was conducted exclusively in French-speaking participants, cross-cultural adaptation and measurement invariance testing across languages, genders, and age groups are needed to ensure broader applicability (63, 64). Taken together, these limitations highlight the need to further examine SO-ACTIV across a wider range of contexts and populations that extend beyond cross-sectional validation. Longitudinal and intervention studies are particularly needed to assess SO-ACTIV’s sensitivity to change over time, and its ability to capture shifts in decisional balance across stages of change. By explicitly embedding DB within the TTM framework, SO-ACTIV provides a theoretically grounded framework for investigating how perceived facilitators and barriers interact with constructs such as self-efficacy and processes of change to influence stage transitions (65, 66). This integrated perspective may ultimately inform the development of more precise, personalized and stage-matched interventions to promote PA among older adults.

## 5 Conclusion

SO-ACTIV is a novel, theory-based instrument with good psychometric properties for assessing decisional balance in physical activity among older adults. By capturing both facilitators and barriers across multiple socioecological levels, it offers a brief yet robust tool for use in research and clinical practice. Its multidimensional structure enables comprehensive profiling of motivational dynamics, thereby supporting the design of tailored stage-matched interventions that address the diverse needs and living contexts of aging populations.

## Data Availability

The raw data supporting the conclusions of this article will be made available by the authors, without undue reservation.
